# Describing the pattern of the COVID-19 epidemic in Vietnam

**DOI:** 10.1080/16549716.2020.1776526

**Published:** 2020-06-26

**Authors:** Van Minh Hoang, Hong Hanh Hoang, Quynh Long Khuong, Ngoc Quang La, Thi Tuyet Hanh Tran

**Affiliations:** Center for Population Health Sciences, Hanoi University of Public Health, Hanoi, Vietnam

**Keywords:** COVID-19, SARS-CoV-2, Vietnam

## Abstract

Given the rapid spread of the COVID-19 pandemic and the huge negative impacts it is causing, researching on COVID-19-related issues is very important for designing proactive and comprehensive public health interventions to fight against the pandemic. We describe the characteristics of COVID-19 patients detected in the two phases of the epidemic in Vietnam. Data used in this paper were mainly obtained from the official database of the Ministry of Health of Vietnam. Descriptive statistics were carried out using Stata 16 software. As of 18 May 2020, the cumulative number of COVID-19 cases detected in Vietnam was 324, 16 cases from 4 cities and provinces in the first phase (during 20 days, 0.8 cases detected per day) and 308 cases from 35 cities, provinces in the second phase (during 76 days, 4.1 cases detected per day). Vietnam has mobilized its entire political system to fight the COVID-19 and achieved some initial successes. We found both similarities and differences between the two phases of the COVID-19 epidemic in Vietnam. We demonstrated that the situation of the COVID-19 epidemic in Vietnam is getting more complicated and unpredictable.

## Background

The progress of COVID-19 has recently been declared by the World Health Organization (WHO) as a public health emergency worldwide [1]. As of 18 May 2020, more than 4.8 million laboratory-confirmed cases had been reported globally. More than 317,000 people have died of COVID-19 [2].

In Vietnam, a middle-income country with a population of more than 96 million people, the first case of COVID-19 was detected on 23 January 2020. As of 18 May 2020, there were 324 confirmed cases throughout the country. Of these cases, 263 have recovered and no death was reported [3]. The COVID-19 epidemic in Vietnam can be described in two phases. The first phase, lasted from 23 January to 13 February 2020, was characterised by detecting and treating 16 cases relating to Wuhan in China. All of these cases recovered and the last patient was discharged on 25 February 2020, thus clearing Vietnam temporarily from COVID-19 for 20 days. The second phase, from 6 March was characterised by the emergence of a further epidemic involving 308 new cases detected in the country. Many of these cases had travelled from Europe, the USA, and other countries, including many Vietnamese nationals. There were also challenges of disease clusters in high population density areas and the potential of silent community transmission. Vietnam mobilised participation from its entire political system to fight the COVID-19 epidemic, employing the principle ‘Early detection, strict quarantine, isolation as well as active treatment’, and achieved some initial successes. The country’s response to the COVID-19 has received acclaim for its effectiveness and transparency [4,5].

Given the rapid spread of the COVID-19 pandemic and the huge negative impacts it is causing, research on COVID-19-related issues is very important for designing proactive and comprehensive public health interventions to fight against the pandemic. In this paper, we describe the characteristics of COVID-19 patients detected in the two phases of the epidemic in Vietnam. The findings from this paper can yield useful insights for developing appropriate intervention actions both in Vietnam and other similar settings in the world.

## Methods

### Data source

Data used in this paper were mainly obtained from the official database of the Ministry of Health of Vietnam (https://ncov.moh.gov.vn/). Additional information was extracted from the Coronavirus Disease (COVID-19) – Statistics and Research website (https://ourworldindata.org/coronavirus#how-long-does-covid-19-last) and provided by provincial centres for disease control in Vietnam. All COVID-19 cases detected before 18 May 2020 were included in our analyses.

### Measurements

In this paper, the following variables were included:

- COVID-19 positive case: A confirmed case of COVID-19 was defined as a positive SARS-CoV-2 test result on high-throughput sequencing or real-time reverse-transcriptase–polymerase-chain-reaction (RT-PCR) assay of nasal and pharyngeal swab specimens. The tests were done by the WHO and MOH approved laboratories in Vietnam [6].
- COVID-19 suspect cases: (1) People developing any symptoms of fever and acute respiratory infections and having a travel history from epidemic areas within 14 days prior to symptom onset; or (2) People having any respiratory symptoms and having any of the following epidemic factors that appear within 14 days prior to symptom onset: (i) close contact with COVID-19 confirmed/suspected cases, or (ii) working in a health facility that treats COVID-19 and contact with COVID-19 patients [7].
- COVID-19 recovered case: A patient who recovered from COVID-19 met three criteria: 1) had no fever for at least three days; 2) had a good general health status; 3) had at least two consecutive negative test results collected at ≥24-h intervals by the RT-PCR method [8]. Continued monitoring and home quarantine process for 14 days after discharge was required for all patients [8].
- The socioeconomic information of the COVID patients, including gender, age, nationality, history of international incoming travel, start date of treatment, date of discharge and duration of treatment, etc.

### Data management and analysis

A research team from Hanoi University of Public Health reviewed and abstracted the data. Data were entered into a computer using Excel software. Descriptive statistics were carried out with means, median, standard deviation and interquartile of continuous variables, count and proportions for categorical variables. To detect the differences in patients’ characteristics between phase one and two, we used Chi-square tests for categorical variables, t-tests or Wilcoxon Rank Sum tests for continuous variables when appropriate. All analyses were conducted using Stata 16 software (Stata Corporation).

### Ethical considerations

The protocol of this study was approved by the Scientific and Ethical Committee in Biomedical Research, Hanoi University of Public Health.

## Results

**Figure 1.**
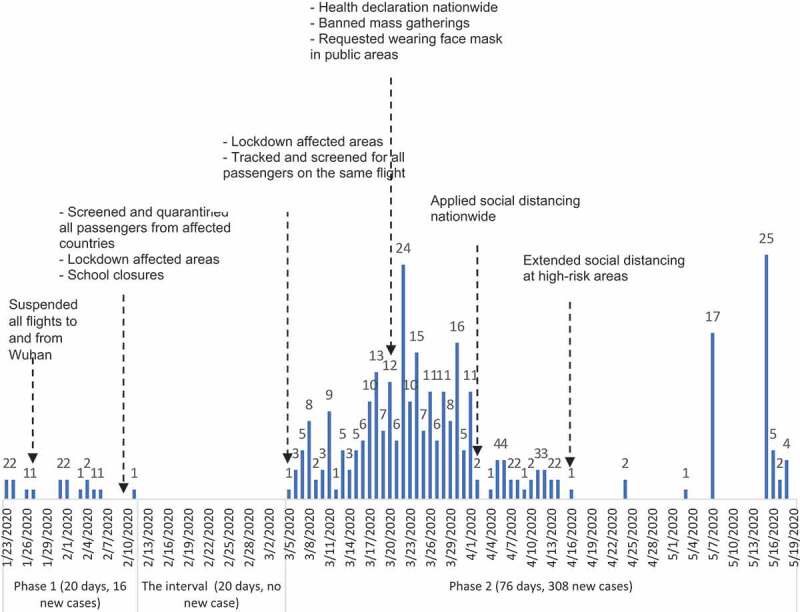
Number of new COVID-19 cases detected per day in Vietnam (as of May 18th, 2020).

As of 18 May 2020, the cumulative number of COVID-19 cases detected in Vietnam was 324, 16 cases from 4 cities and provinces in the first phase (during 20 days, 0.8 cases detected per day) and 308 cases from 35 cities and provinces in the second phase (during 76 days, 4.1 cases detected per day). The highest number of new cases detected per day during the first and the second phase were 2 and 25, respectively (Figure 1).

**Figure 2.**
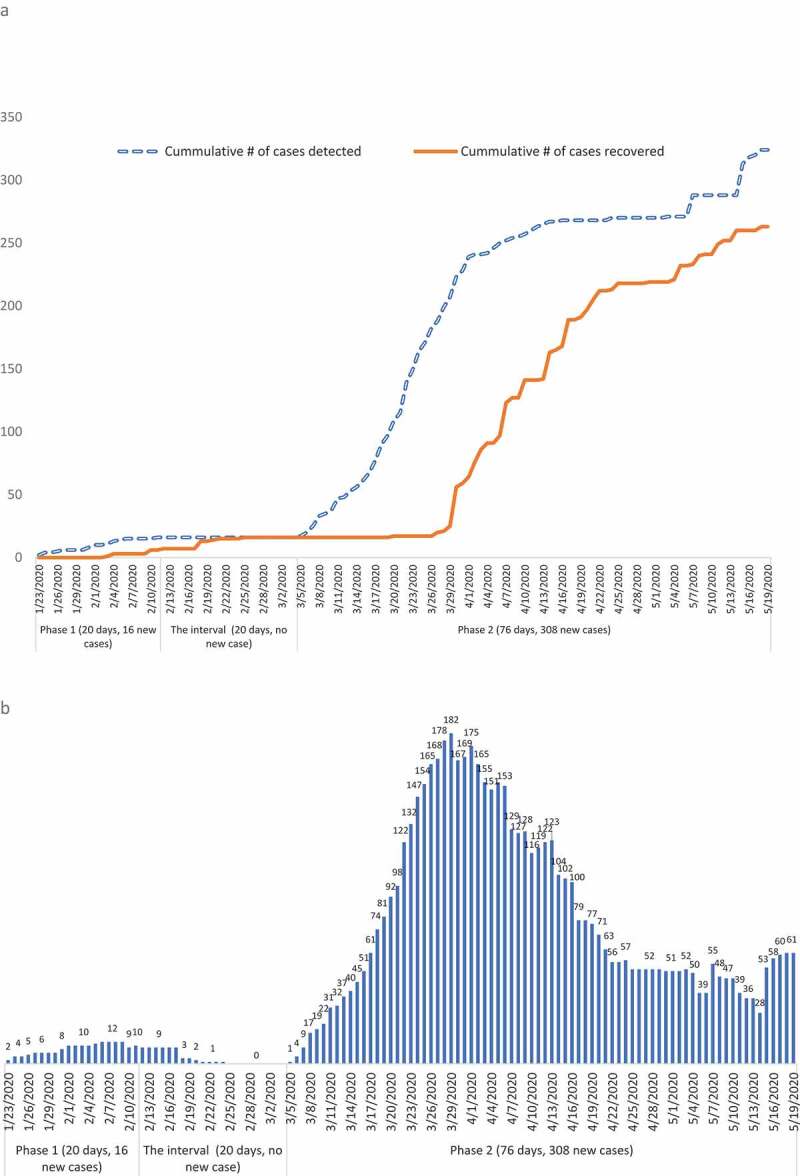
(a) Cumulative number of COVID-19 cases detected and recovered in Vietnam (as of May 18th, 2020). (b) Number of currently positive COVID-19 cases in Vietnam (as of May 18th, 2020).

By 18 May, there were 263 COVID-19 patients fully recovered and discharged (recovery rate of 81.1%), 16 in the first phase and 247 in the second phase (Figure 2(a)), resulting in a peak number of currently positive cases (needing medical treatment on the same day) in the first phase of 12 and in the second phase of 182 (Figure 2(b)).

Table 1 shows the main characteristics of the COVID-19 patients detected during the two phases. The patients detected in the two phases were quite similar in terms of sex distribution, with slightly more females in the first phase. Mean age in both phases was about 35 years. Vietnamese nationals made up more than 80% of cases in both phases, but more than half of all patients had travelled from other countries. However, some differences in the characteristics of the patients detected in the two phases were also observed. As compared to the first phase, the second phase had a higher proportion of patients who had not yet recovered (80.2% vs. 100%, p = 0.048), and longer duration of treatment among recovered cases (19 days vs. 14 days, p < 0.001).

**Table 1.** Characteristics of the COVID-19 patients detected during the two phases.

	The first phase n (%)	The second phase n (%)	
	N = 16	N = 308	
	20 days (Jan 23–Feb 10)	76 days (March 05–May 18)	p
**Sex**			
Female	10 (62.5)	153 (49.7)	0.32
Male	6 (37.5)	155 (50.3)	
**Age**, mean (SD)	35.6 (18.9)	35.2 (15.0)	0.92
**Age**, median (IQR)	29.0 (25.0, 49.5)	30.0 (24.0, 45.0)	0.89
**Age group**			
0–10 years	1 (6.3)	6 (1.9)	0.41
11–20 years	1 (6.3)	36 (11.7)	
21–30 years	8 (50.0)	118 (38.3)	
31–40 years	0 (0.0)	45 (14.6)	
51–60 years	4 (25.0)	80 (26.0)	
60 years and over	2 (12.5)	23 (7.5)	
**Nationality**			
Vietnamese	13 (81.3)	262 (85.1)	0.68
Others	3 (18.8)	46 (14.9)	
**Had traveled from other countries**			
No	8 (50.0)	93 (30.2)	0.095
Yes	8 (50.0)	215 (69.8)	
**Recovered**			
Not yet	0 (0.0)	61 (19.8)	0.048
Yes	16 (100.0)	247 (80.2)	
**Days of treatment among the recovered cases**, median (Min-Max)	14.0 (9–21)	19.0 (5–62)	<0.001

SD: Standard deviation; IQR: Interquartile range

## Discussion

The number of COVID-19 cases detected in Vietnam by 18 May 2020 (324, a rate of 3.4 per million population, and accounting for 0.0067% of 4,848,110 cases reported worldwide) was lower than the rate for Cambodia (8.5 per million) and similar to Laos (3.2 per million), but much lower than the figures of other Asian countries such as Thailand (43.7 per million), the Philippines (119 per million), Malaysia (220 per million), and China (59.6 per million) [9]. The highest number of cases detected per day in Vietnam (24) was lower than the figures reported in other Asian countries such as Cambodia (31), Thailand (188), the Philippines (538), Malaysia (235), and China (14,108) [9].

Vietnam has mobilized its entire political system to fight the COVID-19. Vietnam (as well Laos) ranked number one in the world (score of 100/100) in terms of measures to fight the spread of the COVID-19, stricter than all the other Asian countries [10]. Once the first case of COVID-19 was detected, Vietnam immediately established a National Steering Committee for COVID-19 Prevention and Control under the auspices of the Deputy Prime Minister. Schools in Vietnam were closed from the end of January 2020. The Government also cancelled public events, suspended public transport and imposed travel restrictions. International incoming flights were not allowed from 15 March 2020 and mass quarantining began on 16 March. All the people entering the country from the COVID-19 infected nations and those who had been in direct contact with a confirmed case were put into compulsory quarantine. Vietnam’s layered contact-tracing procedure has also proven critical in battling the virus. Contacts of these people also required self-isolation. Communities, streets or buildings where a case was detected were also quarantined. Vietnam entered a nationwide lockdown from 1 April to 15 2020. The government seriously enforced the lockdown by punishing all cases of violation.

The health system in Vietnam has been proactive and comprehensive in the fight against the epidemic. At the grassroots level, different ‘Rapid Action Teams’ with participation from all the local stakeholders, including health workers (public and private health staffs, retired health professionals and health students, etc.), policemen, soldiers, teachers, representatives of community organizations, and community people, were established to implement comprehensive health education campaigns to raise community knowledge and awareness about COVID-19 and to promote hygienic practices such as using a face mask, washing hands with soap, etc. Financial resources were mobilized from the State budget, donors, charity funds and community people to make ready essential equipment, medicines, and medical supplies. A policy on electronic medical declaration has been applied. Epidemiological investigations, including early detection of infected and suspected cases, close monitoring, supervision of people who had contact with either confirmed or suspected cases, have been actively carried out. The country has provided more than 261.000 tests for COVID-19 to its population [11]. Medical examinations for the elderly, people with chronic diseases, and other vulnerable populations have also been performed. Importantly, confirmed COVID-19 has been mostly treated at local health facilities to avoid unnecessary referrals and burdens to specialised hospitals [12].

We found both similarities and differences between the two phases of the COVID-19 epidemic in Vietnam. The number of COVID-19 cases detected was higher in the second phase reflecting the fact that the number of Vietnamese people living, working and studying abroad who returned home increased in the second phase (82% of the COVID-19 cases who had travelled from other countries were Vietnamese). Moreover, during the second phase, Vietnam had more laboratory centres where standardized testing services were provided and Vietnam expanded the testing services throughout the country [3].

The higher numbers of currently positive cases, the greater absolute number of children and elderly patients in the second phase imply that the needs for medical care and treatments for COVID-19 are increasing in Vietnam. Of note, there were 26 patients with ICU admission or severe illness, accounting for 8% of the total confirmed COVID-19 cases, all of whom were detected in phase two. Depending on the nature of their critical condition, 11 patients were provided with nasal cannulae, 7 needed non-invasive ventilation, 6 needed invasive ventilation, and two received extracorporeal membrane oxygenation [13]. Our findings suggest more special and intensive care (such as specific intensive care (ICU) units and diagnostic lab testing, protocols for management, and adequate equipment for COVID-19, etc.) are likely be needed in the future.

As this was a short-term study using secondary data sources, the results cannot be considered as more than a snapshot of the COVID-19 epidemic in Vietnam. We will need to continue to have more in-depth analyses. However, we have demonstrated that the situation of the COVID-19 epidemic in Vietnam is getting more and more complicated and unpredictable. Although Vietnam achieved initial success in providing timely treatment to the COVID-19 patients as well as in containing the spread of the disease in the community, further proactive and comprehensive actions to tackle the COVID-19 epidemic in this country must be carried out as the global pandemic proceeds.

## Paper context

Vietnam has mobilized its entire political system to fight the COVID-19 and achieved some initial successes. We describe the two phases COVID-19 in Vietnam, by using official database of the Ministry of Health. As of 18 May, the cumulative cases detected were 324, with 0.8 and 4.1 cases per day in the first and second phases., respectively. We suggest that the COVID-19 epidemic in Vietnam is getting more complicated and unpredictable as the global pandemic progresses.
